# Scalable Phosphorus Doping of p‑Type FeS_2_ Microcrystals for Photovoltaic Applications

**DOI:** 10.1021/acsomega.5c07455

**Published:** 2025-11-26

**Authors:** Katriin Reedo, Taavi Raadik, Mare Altosaar, Maris Pilvet, Annaly Gutjuma, Jüri Krustok, Peeter Paaver

**Affiliations:** † Department of Materials and Environmental Technology, 54561 Tallinn University of Technology, Ehitajate Tee 5, 19086 Tallinn, Estonia; ‡ Institute of Ecology and Earth Sciences, 37546 Tartu University, Ülikooli 18, 50090 Tartu, Estonia

## Abstract

Pyrite FeS_2_ is an Earth-abundant semiconductor with the potential to deliver the lowest-cost photovoltaic solutions available today. However, progress has been limited by poor control over doping and surface defect chemistry, leading to consistently low device efficiencies. In this work, we demonstrate for the first time a truly scalable approach to achieve *p*-type conductivity of pyrite microcrystals using phosphorus via the liquid salt growth method. We systematically explore three established doping strategies for semiconductors and identify the successful route involving the use of a FeS + P precursor containing the FeP_4_ phase. Hot probe measurements confirm *p*-type conductivity. Neutral sources such as elemental phosphorus are shown to be thermodynamically unsuitable and fail to induce *p*-type behavior. This study also identifies a phosphorus compound suitable for producing *p*-type FeS_2_ microcrystals, offering a new foundation for the development of pyrite photovoltaic devices.

## Graphical abstract

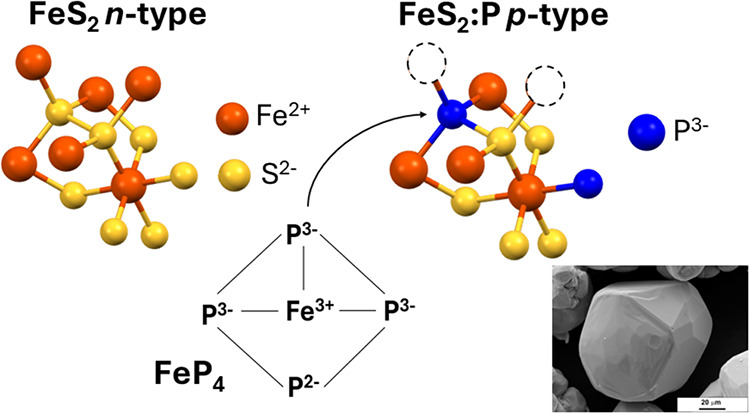

## 1 Introduction

Iron disulfide (FeS_2_) of pyrite structure is an *n*-type semiconductor, typically unintentionally doped via sulfur vacancies.^1,2^ Pyrite (used interchangeably with FeS_2_ in this study) exhibits several key physical properties desirable for photovoltaic absorber materials, including a suitable bandgap of 0.95 eV, a high light absorption coefficient (4 × 10^5^ cm^–1^), and electron mobility of 360 cm^2^ V^–1^ s^–1^ at room temperature.^3–5^ Due to the inexpensive constituent elements, a pyrite solar cell with only 4% efficiency has been projected to match the cost-effectiveness of a 19% efficient silicon-based device.^6^ The low energy requirements for extracting and processing its precursor materials have made FeS_2_ attractive as a potential photovoltaic absorber for extraterrestrial applications, including lunar base power systems.^7^

Despite the long history of research, device efficiencies remain below 3%, primarily due to low open-circuit voltages (*V*_OC_).^8,9^ This limitation arises from the formation of an ultrathin *p*-type inversion layer on the surface of *n*-type pyrite, resulting in a leaky internal junction.^10,11^ This surface inversion effect is particularly pronounced in thin films, where the surface-to-volume ratio is higher than in bulk single crystals. Extensive efforts have focused on understanding this surface inversion and mitigating its effects through chemical and electrochemical etching.^2,3,12^ While trying to avoid the creation of the inverse surface layer is relevant, a potentially more effective strategy is to uniformly dope the crystals, thereby altering their conductivity type from *n*-type to *p*-type and ensuring consistent electronic behavior throughout the whole crystal. Successful *p*-type doping of single-crystal FeS_2_ has only been reported in one study,^13^ in which the authors employed phosphorus (P) doping to synthesize a *p*-type pyrite crystal via chemical vapor transport. Phosphorus was identified as an acceptor approximately 175 meV above the valence band maximum. The study^13^ also reported the solubility limit of P in FeS_2_ at around 100 ppm. This development represents a critical step forward in pyrite photovoltaics and will be advanced further in the current study to develop a scalable method for synthesizing and doping *p*-type pyrite crystals.

In earlier research,^7,14,15^ we employed the molten salt synthesis-growth method to produce FeS_2_ microcrystals, which all showed *n*-type conductivity, as confirmed by hot probe measurements. The molten salt synthesis method enables the production of thousands of individual microcrystals in a single batch. High-quality materials with uniform properties can be successfully synthesized in quantities ranging from just a few grams in sealed quartz ampules to several kilograms in graphite containers.^16^ The microcrystals synthesized in the molten salt can be used for the fabrication of monograin membrane solar cells,^14,17,18^ in which the crystals are fixed within a resin matrix, such as epoxy. Monograin membrane solar cells have distinct advantages, including the separation of absorber crystal synthesis from device assembly and the potential for integration using simple roll-to-roll manufacturing techniques.^16^

An additional benefit of the molten salt synthesis approach is its ability to distribute all the precursors and any impurities uniformly during crystal growth. Our previous findings^15^ revealed that pyrite crystals synthesized by this method exhibited reduced copper impurity concentrations relative to the precursor materials. This purification is driven by thermodynamic equilibrium, which promotes the distribution of impurities between the molten salt and the solid crystal phase. Variations in Fermi level positions and valence band maxima observed with different flux compositions suggest a significant influence of unintentional doping originating from flux-derived impurities.^15^ These observations imply that intentional dopants, such as phosphorus, can also be incorporated into pyrite crystals through the molten salt synthesis-growth process.

In the present work, we develop a novel and scalable technique for producing large volumes of *p*-type FeS_2_ crystals. We explored three different strategies for incorporating phosphorus into pyrite microcrystals and investigated the underlying chemical mechanism. This study is building upon the experimental findings of Voigt et al.^13^ which is the only published study concerning phosphorus-doped *p*-type FeS_2_ single crystals. They report^13^ using the chemical vapor transport (CVT) method for the synthesis of *p*-type material. A key limitation of the CVT approach is its low throughput, typically yielding only a small number of crystals per run. In contrast, by utilizing a molten salt medium containing phosphorus-based dopants, we demonstrate the potential to synthesize and dope thousands of FeS_2_ crystals simultaneously. This liquid-phase growth technique represents a promising route for the scalable production of doped FeS_2_ crystals and may facilitate future mass manufacturing of pyrite-based photovoltaic materials.

## 2 Experimental Section

### 2.1 Materials and Methods

Undoped and doped pyrite crystals were synthesized in sealed quartz ampules using the liquid flux growth method, which is described thoroughly in our previous publications.^14,15^ Iron monosulfide (FeS, 99.9%, Thermo Fischer Scientific) and elemental sulfur (S, 99.999%, Alfa Aesar) were used as precursors for pyrite. Red phosphorus (P, 99.5%, Reahim) was used for doping. The precursors for the synthesis of pyrite were weighed in stoichiometric ratios. Potassium iodide (KI, 99.995%, Acros Organics) was added to the precursor mixture to form a liquid flux phase at the synthesis temperature. The presence of this liquid phase facilitates the growth of individual FeS_2_ microcrystals. To achieve optimal conditions, KI was added in an amount such that the volume of the molten KI approximately matched the volume of the solid FeS_2_ precursors. This ensures that the liquid phase fills the voids between solid particles, promoting uniform crystal growth and enabling repulsive interactions between forming FeS_2_ crystals, which helps prevent agglomeration. The precursors (FeS and S) and KI were mixed and inserted into quartz ampules. The ampules were degassed in a dynamic vacuum, sealed in flame, and placed into a chamber furnace. The ampules were heated to 690 °C, a little bit over the melting point of KI (681 °C)^19^ and kept at 690 °C for 10 days. Pyrite crystals grow in these conditions by the Ostwald ripening mechanism.^20^ The synthesis-growth lasted for 10 days, to give enough time to form FeS_2_ microcrystals that are sufficiently large for our application. For the fabrication of monograin membranes, each microcrystal should fall in the diameter range of 40–150 μm. The crystals are then sieved into narrow granulometric fractions. Under our synthesis conditions, it typically^7,14,15^ takes about 10 days to produce a batch in which a significant fraction of the FeS_2_ crystals meet this size requirement. During the high-temperature synthesis, the pressure inside the ampules is primarily generated by sulfur that has not yet reacted with FeS. The vapor pressure of sulfur at 690 °C is high, at around 5000 Torr. After the synthesis, KI is removed from the FeS_2_ crystals by leaching in deionized water.

Several strategies were applied to dope pyrite microcrystals with phosphorus. The only previously published study on phosphorus doping of pyrite^13^ served as the basis for the first experiment. In that study, FeS_2_ crystals were synthesized via chemical vapor transport using FeS_2_ powder and red phosphorus. Building on this approach, we designed a two-chamber quartz ampule system, drawn in Figure 1(1). The chambers were connected by a narrow neck that allowed the transfer of phosphorus vapor. FeS_2_ crystals presynthesized as described before were placed in one chamber, while a few milligrams of red phosphorus were placed in the other. The ampules were evacuated, sealed, and heated in a furnace at 500 °C for 48 h. The vapor pressure profiles of sulfur and phosphorus are similar, and the vapor pressure of phosphorus at 500 °C is approximately 3000 Torr, assuring effective material transport under these conditions.

**Figure 1:**
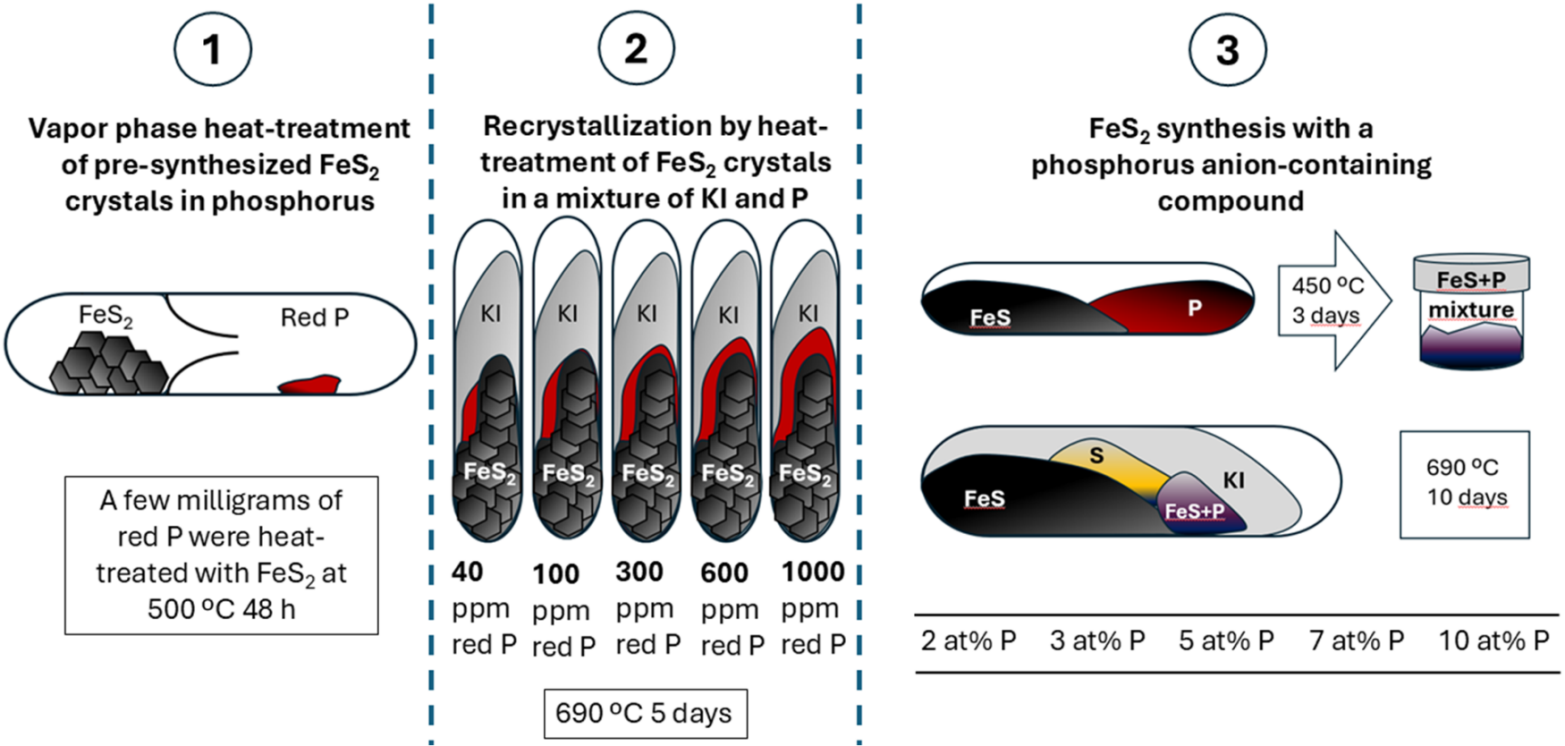
Schematic drawing of the three adopted doping treatments: (1) in the vapor phase, (2) during recrystallization, and (3) during synthesis of pyrite crystals.

Next, red phosphorus was mixed with potassium iodide and employed as a flux during the molten-phase recrystallization of presynthesized pyrite microcrystals. For the recrystallization, previously synthesized FeS_2_ material was placed in quartz ampules. A potassium iodide and phosphorus mixture was prepared separately by adding P to KI in the amount to yield 1000 ppm P in KI. This 1000 ppm mixture was diluted with pure KI to yield the final concentrations and was added to the FeS_2_ crystals to yield 40, 100, 300, 600, and 1000 ppm phosphorus, on a molar basis, in each ampule, respectively. This concentration is equivalent to 0.004, 0.01, 0.03, 0.06, 0.1 atom % P. The ampules were degassed, sealed, and heated at 690 °C for 5 days. This process is visualized in Figure 1(2).

The third approach to incorporating phosphorus into pyrite involved using a self-synthesized iron sulfide–phosphide (hereafter FeS + P) precursor. This precursor was prepared by mixing equimolar amounts of FeS and red phosphorus, followed by heat treatment in a sealed quartz ampule at 450 °C for 3 days. After heating, the FeS + P mixture was extracted and stored for subsequent use. For the synthesis of phosphorus-doped pyrite, this precursor was combined with FeS and sulfur in a quartz ampule. The amounts were calculated to yield stoichiometric FeS_2_ with phosphorus concentrations of 2, 3, 5, 7, and 10 atomic percent (atom %) relative to sulfur in the final crystals. Potassium iodide flux was added as described previously for the undoped samples. The visual representation is shown in Figure 1(3). The degassed and sealed ampules were then heated at 690 °C for 10 days to complete the synthesis.

A mixture of iron and phosphorus (hereafter Fe + P) was synthesized in the same way as described above and used as an alternative to the FeS + P mixture to synthesize and dope FeS_2_ crystals in a parallel experiment. Figure 1 represents a schematic depiction of the three doping strategies explored in this study.

### 2.2 Analytical Techniques

Materials were analyzed by different methods to evaluate the success of each phosphorus treatment and to understand the possible chemical route of phosphorus incorporation into pyrite microcrystals. The phase composition was analyzed by Raman spectroscopy, using a Horiba LabRam HR800 spectrometer equipped with a multichannel CCD detection system in the backscattering configuration. 532 nm laser line with a spot size of 5 μm was applied for excitation. X-ray diffraction (XRD) patterns were recorded on a Rigaku Ultima IV diffractometer with Cu Kα radiation (λ = 1.5406 Å). PDXL 2 software was used to derive crystal structure information from the recorded XRD data.

The conductivity type of crystals was determined by the hot probe method. For this technique, a sample crystal is placed between two contacts. One contact or probe is heated, thermally exciting the charge carriers in the vicinity of the hot probe. Carriers move by diffusion from the hot probe to the “cold” probe, which stays at room temperature. The type of majority carriers defines the electrical potential sign in the multimeter.^21^

The chemical composition of crystals was assessed by energy dispersive X-ray spectroscopy (EDX) using a Bruker Esprit 1.8 system. The EDX measurements were taken from the cross-section (bulk) of materials, from at least 8 individual crystals of each sample. The measurement limit of the EDX system is 0.1 atom %.

Inductively coupled plasma mass spectroscopy (ICPMS) was used to determine the level of impurities in crystals. 0.1 g of solid samples were dissolved in a mixture of 8 mL of HNO_3_ and 2 mL of H_2_O_2_ using an Anton Paar Multiwave PRO microwave digestion system. Samples were digested at 230 °C at pressures between 45–50 bar. The sample solutions were diluted with 2% HNO_3_. Concentrations of impurity elements were measured using Agilent 8800 ICPMS/MS. Indium was used as an internal standard element added online via mixing T and NIST 1643f, which were used as references for quality control.

The morphology of different crystals was evaluated using high-resolution scanning electron microscopy (SEM) Zeiss ULTRA 55.

## 3 Results and Discussion

### 3.1 Vapor Phase Heat-Treatment of Pre-Synthesized FeS_2_ Crystals in Phosphorus

Phosphorus incorporation in pyrite crystals was first carried out via vapor-phase treatment. Post-treatment analysis revealed that P reacted with FeS_2_, resulting in the formation of two distinct phases. Part of the material remained in the FeS_2_ phase; however, a significant portion of the pyrite crystals exhibited cracking or fragmentation, as shown in Figure 2a–d, where the different phases are evident in the SEM backscattered electron images. Energy-dispersive X-ray spectroscopy (EDX) indicated a high phosphorus composition, 20–25 atom % P, within the fragmented phase; the EDX spectra are shown in Supporting Information 1. Raman spectroscopy results are shown in Figure 2a–d. The phosphorus-rich, fragmented material showed characteristic peaks of cubic FeS_2_ at 343, 350, 379, and 431 cm^–1^,^22,23^ along with additional peaks at 247 and 279 cm^–1^ corresponding to iron thiophosphide phases such as Fe_2_P_2_S_6_ or FePS_3_.^24^ The Raman peak at 343 cm^–1^ corresponds to the E_g_ Raman mode, where the sulfur atoms are displaced perpendicular to the axis of the sulfur–sulfur bond. A weak Raman peak at 350 cm^–1^ reflects the *T*_g_ phonon mode, which reflects the in-phase and out-of-phase stretching vibrations of the sulfur dimer S_2_. The strong Raman peak at 379 cm^–1^ belongs to the A_g_ mode, which corresponds to the same stretching vibrations as the *T*_g_ mode. The Raman peak at 430 cm^–1^ is also attributed to the *T*_g_ phonon mode.^25,26^

**Figure 2:**
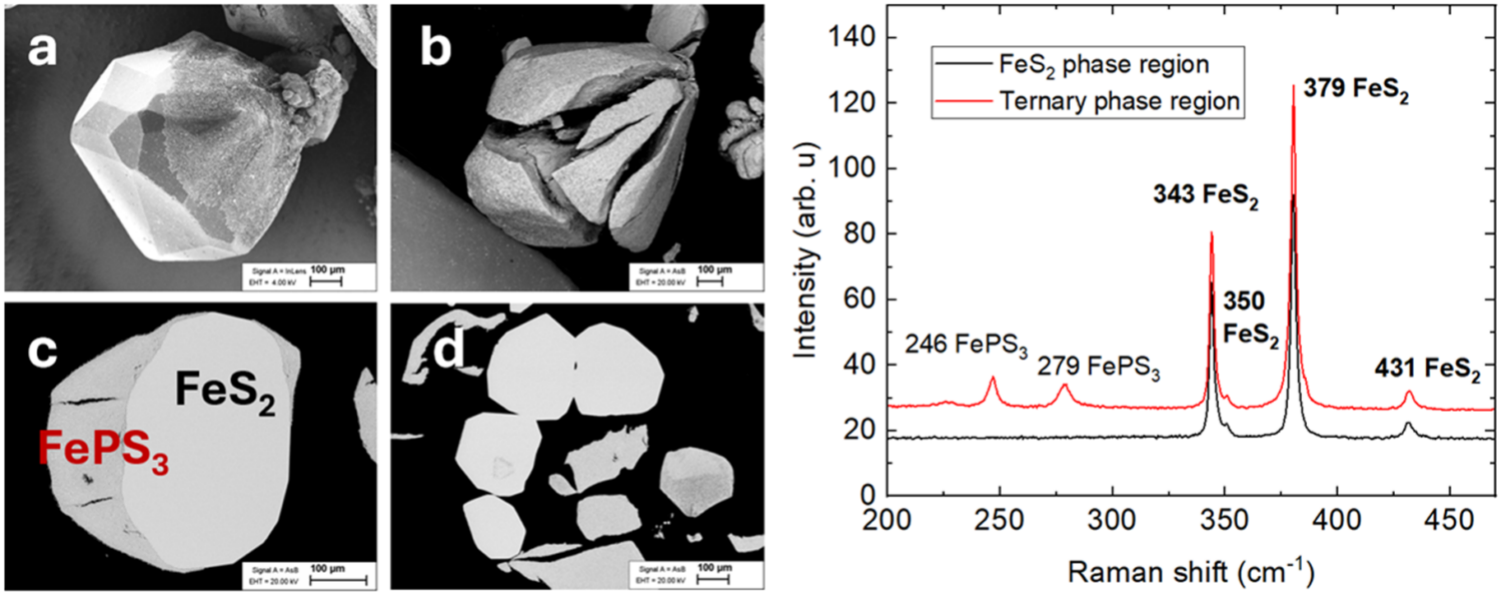
Left: SEM images of the FeS_2_ microcrystals after heat treatment in P vapor atmosphere. (a, b) images of the surface, (c, d) images of the cross-section. Right: Raman spectra of the pyrite crystals’ cross-section after treatment in the phosphorus vapor atmosphere.

The leafy, needle-like structure of FePS_3_ is also seen inside the cracks of the fragmented crystals. The reason for the cracking and fragmentation is likely due to the layered structure of the FePS_3_ phase and its different density compared to pyrite.

All the performed hot probe measurements on different crystals confirmed *n*-type conductivity as the majority carrier type. Thus, it was concluded that phosphorus vapor treatment was not suitable for doping FeS_2_ crystals with P.

### 3.2 Recrystallization by Heat-Treatment of FeS_2_ Crystals in a Mixture of KI and P

In the second series, increasing amounts of red phosphorus (10 to 1000 ppm P relative to presynthesized FeS_2_, on molar basis) were mixed with the flux salt (KI) to perform phosphorus treatments on presynthesized pyrite crystals. Unlike the prior series, these treatments were conducted in a molten KI flux medium at elevated temperatures, facilitating recrystallization of the pyrite microcrystals and enabling phosphorus incorporation into the FeS_2_ lattice in the recrystallization-growth process. Post-recrystallization, SEM analysis revealed no cracks or secondary phases, even for the highest P concentration, as shown in Figure 3. Raman spectra with sharp and narrow peaks at 343, 380, and 431 cm^–1^ confirm the single pyrite phase,^22,23^ as shown in Figure 4.

**Figure 3:**
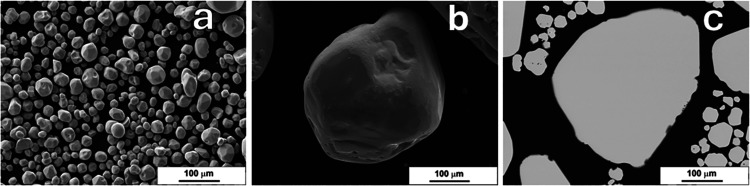
SEM images of FeS_2_ microcrystals recrystallized with 1000 ppm phosphorus: (a, b) surface morphology and (c) cross-section of a single crystal.

**Figure 4:**
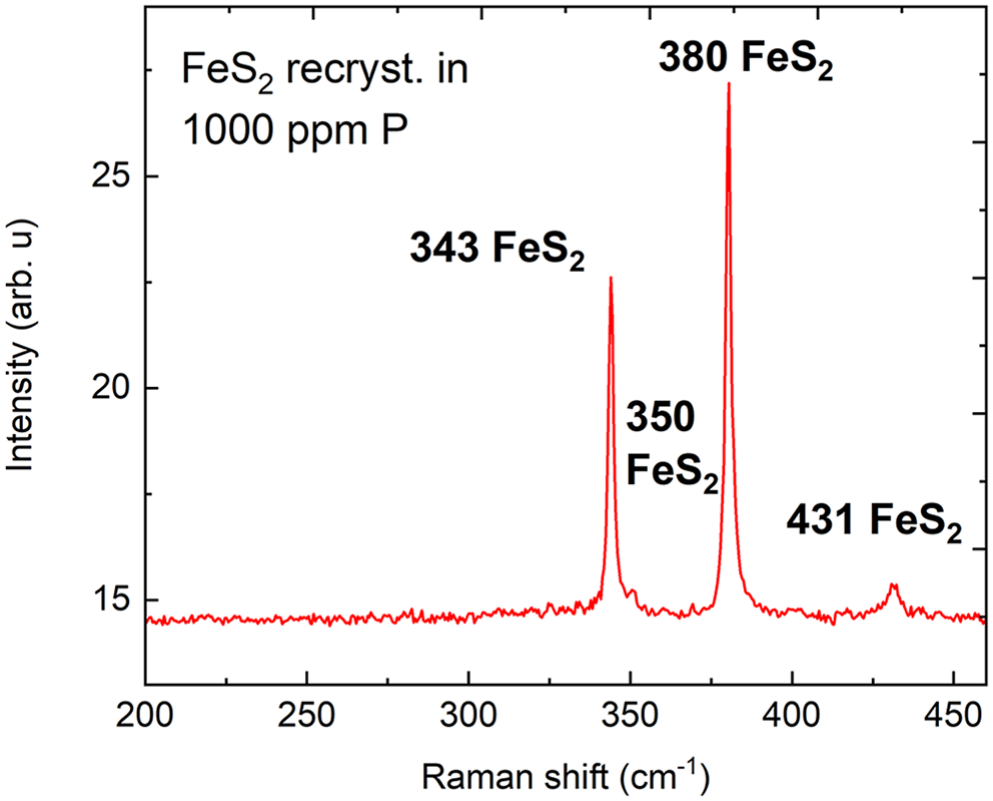
Raman spectrum of the FeS_2_ material recrystallized in the presence of phosphorus at a 1000 ppm level.

However, phosphorus was not detected by the EDX analysis, even in samples treated with 1000 ppm P. This fact suggests that phosphorus was present either below the EDX detection limit or that elemental phosphorus was preferentially dissolved in KI, thereby limiting diffusion into the pyrite lattice. As derived from the hot probe measurements, the recrystallized material’s conductivity remained *n*-type and was not changed after the treatment.

ICPMS analysis data of pyrite microcrystals recrystallized in KI with added phosphorus are presented in Table 1. The determined P levels in pyrite crystals remained below the specific detection accuracy of the ICPMS instrument. Notably, the undoped pyrite exhibited nearly twice the phosphorus concentration (59 ppm) compared to the doped samples. This suggests that phosphorus is predominantly extracted from solid crystals during recrystallization in the liquid flux, due to the distribution of P between solid and liquid phases. The purification phenomenon in the molten salt was also reported in one of our previous works.^15^

**Table 1:** ICPMS Analysis Data of Pyrite Microcrystals Recrystallized in Ki with Added Phosphorus

intended P level in FeS_2_		analyzed P content in FeS_2_/ppm (molar basis)	measurement error ±
0.004 atom %	40 ppm	27^a^	0.22
0.01 atom %	100 ppm	29^a^	0.40
0.03 atom %	300 ppm	30^a^	0.08
0.06 atom %	600 ppm	32^a^	0.22
0.1 atom %	1000 ppm	39	0.72
undoped, not treated		59	2.27

a Below detection accuracy.

The reason for the lack of phosphorus incorporation may be the oxidation state of phosphorus in the used dopant. In Chapter 3.1, we saw that sulfur, as a strong oxidizer, oxidized phosphorus to the P^3+^ oxidation state and, as a result, FePS_3_ formed. In the current chapter, another limiting phenomenon is revealed. The added P amounts were relatively small, and the determined phosphorus contents (Table 1) were below the undoped and untreated material (P as residual impurity, likely originating from the FeS precursor). This fact shows that the liquid phase of KI extracted phosphorus from the solid crystals via the distribution of P between the liquid and solid phases.

Building on the results of the first two methods, we discovered that postsynthetic diffusion of phosphorus into FeS_2_ crystals is not possible. Phosphorus either reacts with pyrite, as shown in Chapter 3.1, or is lost between the solid and liquid phases when added in insufficient quantities. Therefore, phosphorus must be incorporated into the pyrite structure during the crystal growth process.

According to *The Chemistry of Imperfect Crystals* by F. A. Kröger,^27^ successful doping of pyrite requires phosphorus atoms to substitute for sulfur ions in the lattice. Given that sulfur exists as S^2–^, a phosphorus ion with the same charge (P^2–^) would not alter the defect chemistry. To induce iron vacancies and promote *p*-type conductivity, phosphorus must be incorporated as P^3–^. Therefore, an effective dopant must be a phosphorus-containing compound in which phosphorus carries a negative charge and remains thermally and chemically stable at the synthesis temperature.

### 3.3 FeS_2_ Synthesis with a Phosphorus Anion-Containing Compound

Two new phosphorus precursors were synthesized to produce a stable compound containing phosphorus in an anionic state. Mixtures of FeS and P (FeS + P), and Fe and P (Fe + P), were prepared and heated in quartz ampules as outlined in Section 2. These precursors served as phosphorus sources in two parallel series of pyrite microcrystal syntheses. The rationale was that prereacting FeS or Fe with phosphorus would (a) prevent the formation of the layered FePS_3_ phase observed in vapor-phase doping (Chapter 3.1) by stabilizing phosphorus in a compound, and (b) promote incorporation of phosphorus in a favorable oxidation state on sulfur sites. The FeS + P precursor was added to the pyrite precursor mixture in quantities corresponding to 2, 3, 5, 7, or 10 atom % P per sulfur in FeS_2_. These relatively high phosphorus loadings were chosen to account for the potential dissolution of P in liquid potassium iodide. The nominal phosphorus contents and resulting compositions after the synthesis process were measured by EDX and are summarized in Table 2. Despite the high phosphorus input, the resulting pyrite crystals contained very low amounts of phosphorus, often below the EDX detection limit. The EDX mapping results are shown in Supporting Information 2.

**Table 2:** EDX and Conductivity Type Data of Pyrite Microcrystals Synthesized in 2–10 atom % P

concentration of P in the FeS_2_ synthesis (atom %)	concentration of P in the obtained material (atom %)	conductivity type, determined by hot probe
2	0	*n*-type
3	0	*n*-type
5	0	*p*-type
7	0–1.3	*p*-type
10	0–1.4	*p*-type

Materials that were synthesized with 5, 7, or 10 atom % phosphorus exhibited *p*-type conductivity, while those with 2 or 3 atom % phosphorus remained *n*-type. This shows that sufficient phosphorus incorporation, particularly in a chemically available form, can effectively alter the conductivity type of pyrite crystals.

The morphological comparison of the pyrite materials is shown in Figure 5. It was found that the sample synthesized with 10 atom % P (Figure 5c,d) exhibited minor surface cracks. In contrast, no such fragmentation was observed in the samples synthesized with 2, 3, 5, or 7 atom % P, which all show similar morphology, shown in Figure 5a,b. The cracking may result from the formation of a ternary FePS_3_ phase. In the first part of this study (where presynthesized pyrite crystals were treated in P vapor), we observed that excess phosphorus led to the formation of FePS_3_–a layered material with a lower density than FeS_2_. The coexistence of these two phases, with their distinct structural and physical properties, can induce internal stress during synthesis or cooling, leading to cracking and fragmentation. Uniformly composed microcrystals with smooth surfaces are required for the fabrication of monograin membranes; thus, the material synthesized with 10 atom % P appears unsuitable for further application. Considering the *p*-type conductivity and minimal morphological changes, the FeS_2_ synthesized with 5 atom % P was selected for subsequent analyses and experiments. The uniformity of materials synthesized by the liquid salt synthesis method is discussed further in Supporting Information 3.

**Figure 5:**
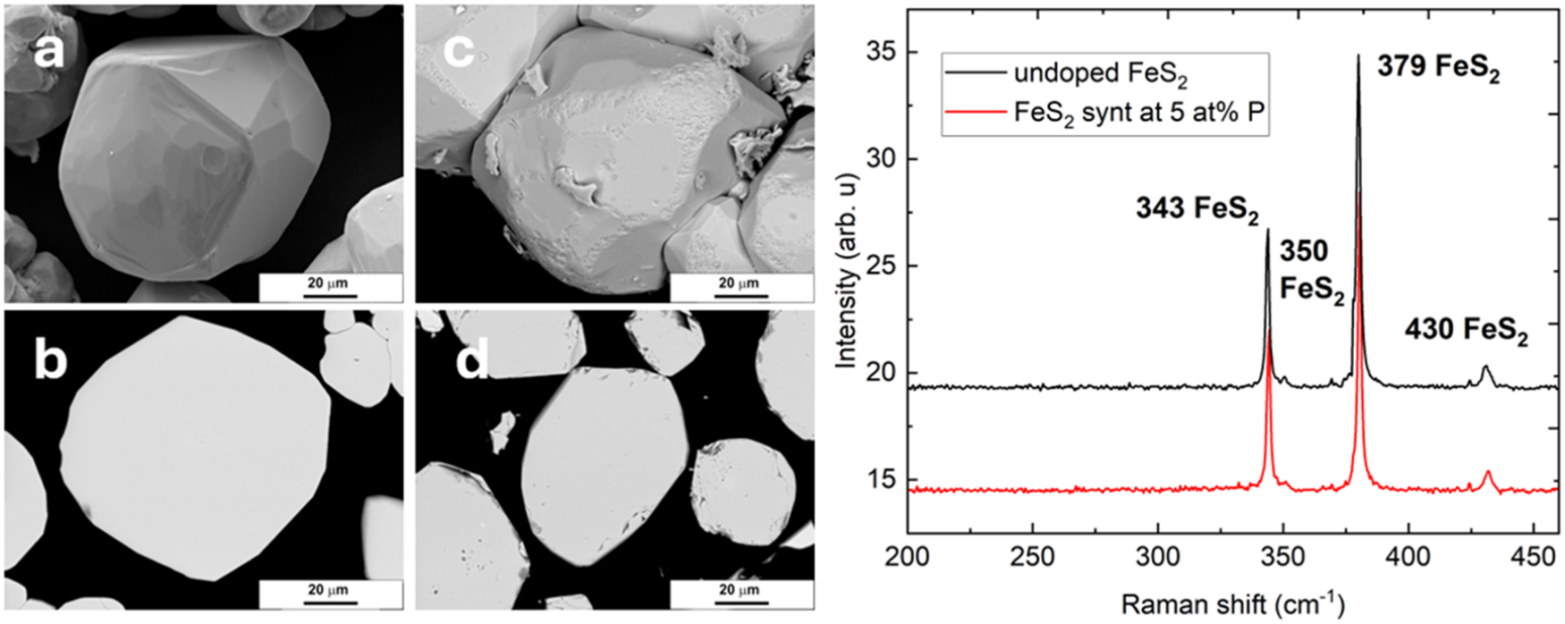
Left: SEM images of the FeS_2_ materials synthesized and doped using a previously prepared FeS + P mixture. FeS_2_ crystals were synthesized with (a, b) 5 atom % P; and (c, d) 10 atom % P. Right: Raman spectra of the *n*- and *p*-type FeS_2_ materials. Black line: *n*-type and undoped FeS_2_. Red line: *p*-type FeS_2_, synthesized with 5 atom % P, using the FeS + P precursor.

The phase composition of the materials was analyzed using Raman spectroscopy and X-ray diffraction. The Raman spectra of the undoped *n*-type materials are compared to those of the phosphorus-doped *p*-type material (synthesized with 5 atom % P) in Figure 5. No additional phases beyond the pyrite phase were identified.

Figure 6 compares the X-ray diffractograms of the undoped *n*-type pyrite and the phosphorus-doped *p*-type sample synthesized with 5 atom % P. In addition to the characteristic pattern of the pyrite phase, XRD revealed additional signals corresponding to FePS_3_, a secondary phase previously identified in this study. FePS_3_ was detected only by XRD and not by Raman spectroscopy, likely due to its low concentration. While Raman analysis probes small (∼5 μm) localized areas, XRD integrates over a larger sample area, enhancing the detection of minor phases, such as FePS_3_, which is found in very low amounts between the individual FeS_2_ microcrystals. The reaction pathway leading to the formation of the layered FePS_3_ phase is detailed in Supporting Information 4. A comparative table of all the doping techniques and their outcomes is shown in Supporting Information 5.

**Figure 6:**
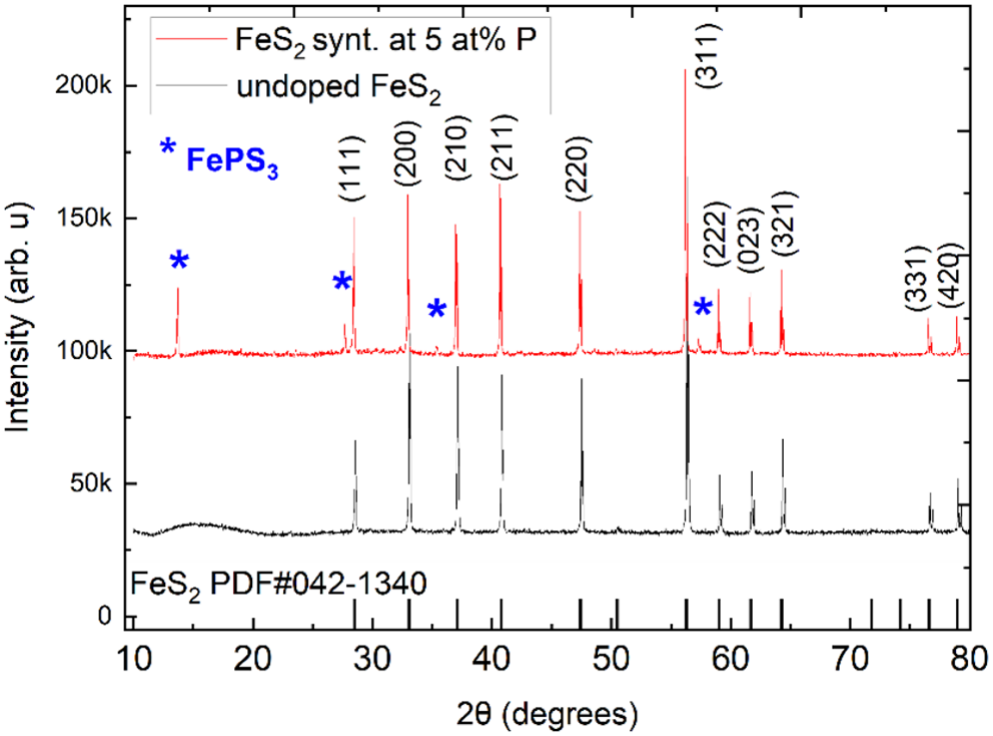
XRD results of the doped and undoped pyrite microcrystals, synthesized and doped by the flux growth method. Black pattern: undoped pyrite crystals. Red pattern: pyrite crystals synthesized with 5 atom % P using the FeS + P precursor.

The Fe + P precursor, synthesized from elemental iron and phosphorus, was used to prepare P-doped pyrite with a target concentration of 5 atom % phosphorus. This was carried out by combining Fe + P, FeS, S, and KI, followed by heating at 690 °C as previously described. SEM and Raman analyses revealed no significant differences in morphology or phase composition compared to undoped materials. However, all samples exhibited *n*-type conductivity, indicating that phosphorus doping was ineffective. As a result, these findings are not discussed further.

### 3.4 Proposed Phosphorus Compound and Doping Mechanism for *p*-type FeS_2_

Phosphorus is well-known for its ability to adopt multiple oxidation states and form various iron phosphide compounds.^28,29^ Identifying the specific compound that enables effective doping of pyrite and changes its conductivity is critical for improving the reproducibility of this doping method.

The observation that the use of Fe + P precursor does not lead to *p*-type doping, whereas the FeS + P precursor yields *p*-type pyrite, suggests that a specific compound that allows phosphorus incorporation in pyrite is present in the latter. This compound, absent in the Fe + P system, likely facilitates phosphorus incorporation into pyrite in a chemically compatible form and promotes hole generation, thereby inducing a transition from *n*-type to *p*-type conductivity. XRD analysis was performed on both types of precursor mixtures to identify the relevant phases present in each precursor system. The phase compositions are shown in Figure 7 and in Table 3. The FeS + P precursor was found to contain P_4_S_6_, pyrrhotite (Fe_0.893_S),^30^ and FeP_4_. In contrast, the Fe + P precursor contained elemental phosphorus, FeP, and Fe_2_P. Among these, P_4_S_6_ and FeP_4_ are the possible candidates for P incorporation in FeS_2_. P_4_S_6_ contains phosphorus in the 3+ oxidation state,^31^ chemically unsuitable for occupying S^2–^ sites.^32^ However, the P_4_S_6_ phase might be responsible for the creation of the FePS_3_ minority phase that was recognized by the XRD measurements of the *p*-type pyrite material, shown in Figure 6. The possible reaction pathway is brought in Supporting Information 4.

**Figure 7:**
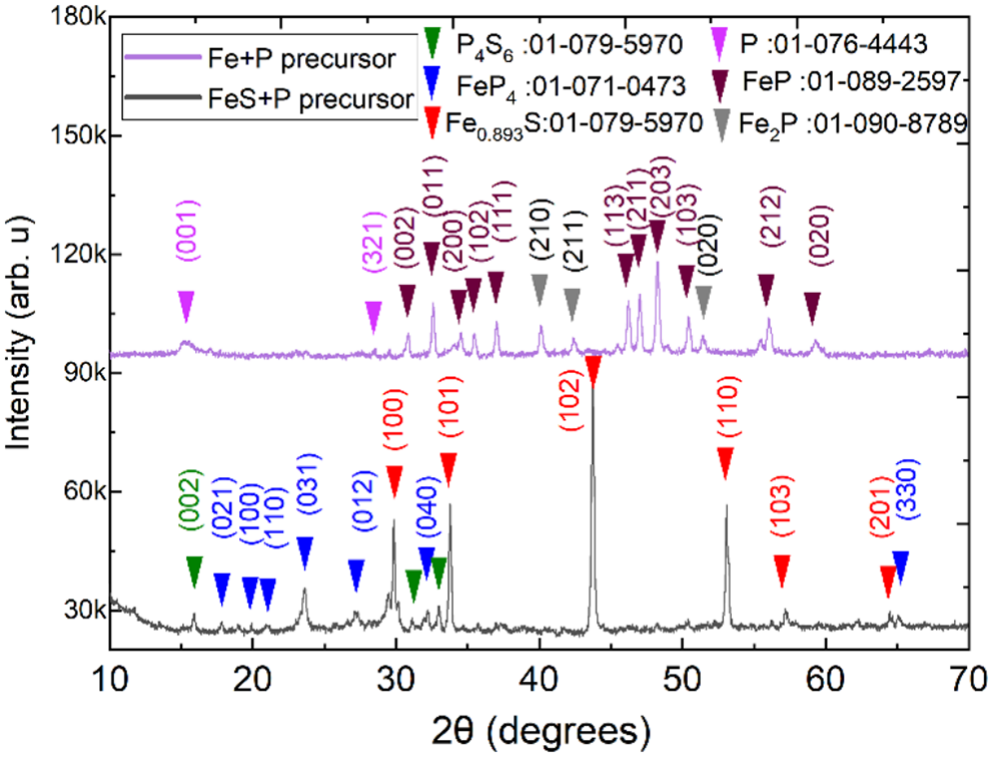
XRD results of the two different phosphorus precursors. Black pattern: precursor mixture prepared from FeS and P. Purple pattern: the precursor mixture prepared by heating elemental Fe and P.

**Table 3:** Summary of the Phosphorus Precursors Phase Composition, Based on the XRD Results

used precursor	compounds present, determined by XRD	quantity in the mixture (%)	charge of the phosphorus ion
Fe + P mixture, heated at 450 °C	elemental phosphorus	9	0
	FeP	72	2-; 3-^a^
	Fe_2_P	19	3-^a^
FeS + P mixture, heated at 450 °C	P_4_S_6_	2	3+
	pyrrhotite (Fe_0.893_S)	8	n/a
	FeP_4_	90	2-; 3-

a In this case, the phosphorus is bound to iron by double and triple bonds, which have higher energy and are not broken under our synthesis conditions.

The FeS + P precursor also contained FeP_4_, which is a distinct iron phosphide phase with the Fe atom surrounded octahedrally by six P atoms.^33^ Phosphorus atoms are arranged as tetrahedra with either two P atoms and two Fe atoms, or three P atoms and one Fe atom. In this configuration, phosphorus is found in the 3- and 2- anionic states.^33^

The FeP and Fe_2_P phases in the Fe + P precursor also host P in 3- and 2- charge, but the phosphorus and iron are bound with double and triple bonds with dissociation energies up to 2 or 3 times the Fe–P single bond.^34,35^ The synthesis conditions (temperature and pressure) for FeS_2_ synthesis in the liquid phase do not allow for the breaking of higher energy Fe–P multinary bonds, making the FeP_4_ phase the only available compound for phosphorus doping during the synthesis of pyrite microcrystals. The Fe–P–S phases that are not participating in the doping or synthesis of pyrite dissolve in the liquid salt flux and are removed after the synthesis process.

The proposed doping mechanism is based on the theory of Kröger.^27^ Doping takes place in pyrite when phosphorus atoms substitute for sulfur ions within the pyrite lattice. Since sulfur carries a 2- charge and phosphorus in our setup a 3- charge, phosphorus accepts more electrons than sulfur. This substitution leads to the formation of iron vacancies, which act as *p*-type acceptor defects in pyrite. When FeP_4_ is used as a doping compound, four P atoms will substitute for four S atoms, at the same time introducing only one Fe atom. This mechanism generates three Fe vacancies in the pyrite lattice. Phosphorus does not occupy sulfur vacancy sites–the generation and elimination of sulfur vacancy defects are governed by thermal treatments and sulfur vapor pressure.^27,36^ Our findings indicate that even under saturated sulfur pressure at 690 °C, sulfur vacancies are not fully suppressed, nor are iron vacancies effectively induced. A comprehensive understanding of synthesis conditions and doping strategies is essential for advancing the use of *p*-type pyrite crystals as the absorber of photovoltaic devices and for enabling the development of pyrite-based solar cells.

## 4 Conclusions

This study investigated three approaches to achieve *p*-type doping of FeS_2_ (pyrite) crystals. The first involved postsynthesis heat treatment of FeS_2_ crystals in a phosphorus vapor atmosphere. The second approach utilized high-temperature recrystallization of FeS_2_ in a mixture of molten KI and elemental P. Both methods, however, resulted in *n*-type FeS_2_. The third strategy, synthesizing pyrite crystals in a liquid salt medium using a phosphorus-containing precursor, proved successful, yielding *p*-type FeS_2_. This method represents the first scalable approach for phosphorus doping of FeS_2_ crystals. The phosphorus precursor was prepared by reacting FeS with elemental P in an evacuated quartz ampule at 450 °C. XRD analysis confirmed that the resulting precursor mixture contained FeP_4_, which was identified as the only effective phosphorus compound enabling incorporation into the FeS_2_ lattice during synthesis. The resulting doped material was characterized by Raman spectroscopy and XRD, both confirming the formation of the pyrite phase. Although the phosphorus concentration was below the EDX detection limit of ∼0.1 atom %, hot-probe measurements indicated a clear conductivity type inversion from *n*-type to *p*-type.

A mechanism for phosphorus incorporation into the pyrite lattice is proposed based on theoretical considerations: FeP_4_ facilitates the substitution of sulfur sites by phosphorus atoms in the pyrite lattice. For every four phosphorus atoms incorporated, one iron site is occupied, leading to the formation of three iron vacancies, which act as acceptors and enable hole conduction. These findings provide a foundation for future development of photovoltaic devices based on *p*-type FeS_2_, including potential applications in homojunction solar cells.

## Supplementary Material



## Notes

EDX spectra and mapping results of FeS_2_ materials discussed in this study, a discussion on the uniformity of the material across larger batches, the reaction pathway for the creation of the layered FePS_3_ phase, and a comparison table of the used doping techniques (PDF)
