# Supplementary material for: Scalable Phosphorus Doping of p‑Type FeS2 Microcrystals for Photovoltaic Applications
Source: ACS Omega. 2025 Nov 26;10(48):58869–76. doi: 10.1021/acsomega.5c07455 (PMC12771458; doi:10.1021/acsomega.5c07455)
Supplement: Supplementary file 1 [file ao5c07455_si_001.pdf]

Supporting information

Scalable Phosphorus Doping of *p*-Type FeS_2 Microcrystals for Photovoltaic Applications

Katriin Reedo*[a], Taavi Raadik[a], Mare Altosaar[a], Maris Pilvet[a], Annaly Gutjuma[a], Jüri Krustok[a],

Peeter Paaver[b]

a. Department of Materials and Environmental Technology, Tallinn University of Technology, Ehitajate Tee 5, 19086, Tallinn, Estonia

b. Institute of Ecology and Earth Sciences, Tartu University, Ülikooli 18, 50090, Tartu, Estonia

* Email: katriin.reedo@taltech.ee

S1 EDX spectra of FeS_2 materials discussed in this study

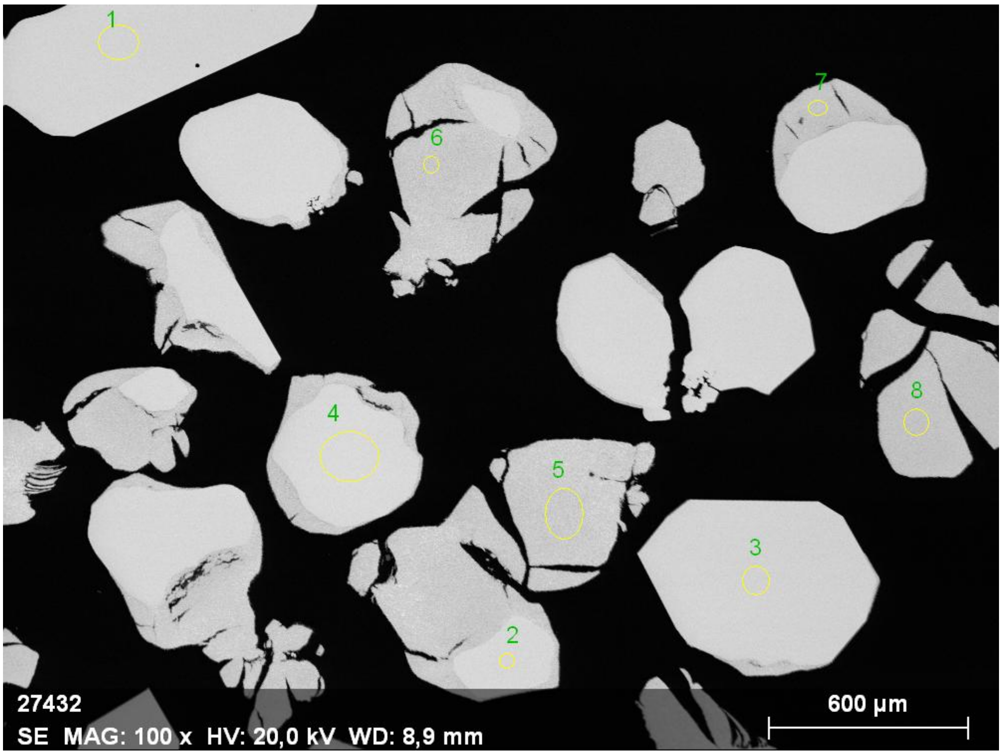

Figure S1. The EDX data were recorded from the polished cross-sections of the FeS_2 microcrystals after heat treatment in the phosphorus vapor. Numbers 1-8 mark the measurement areas.

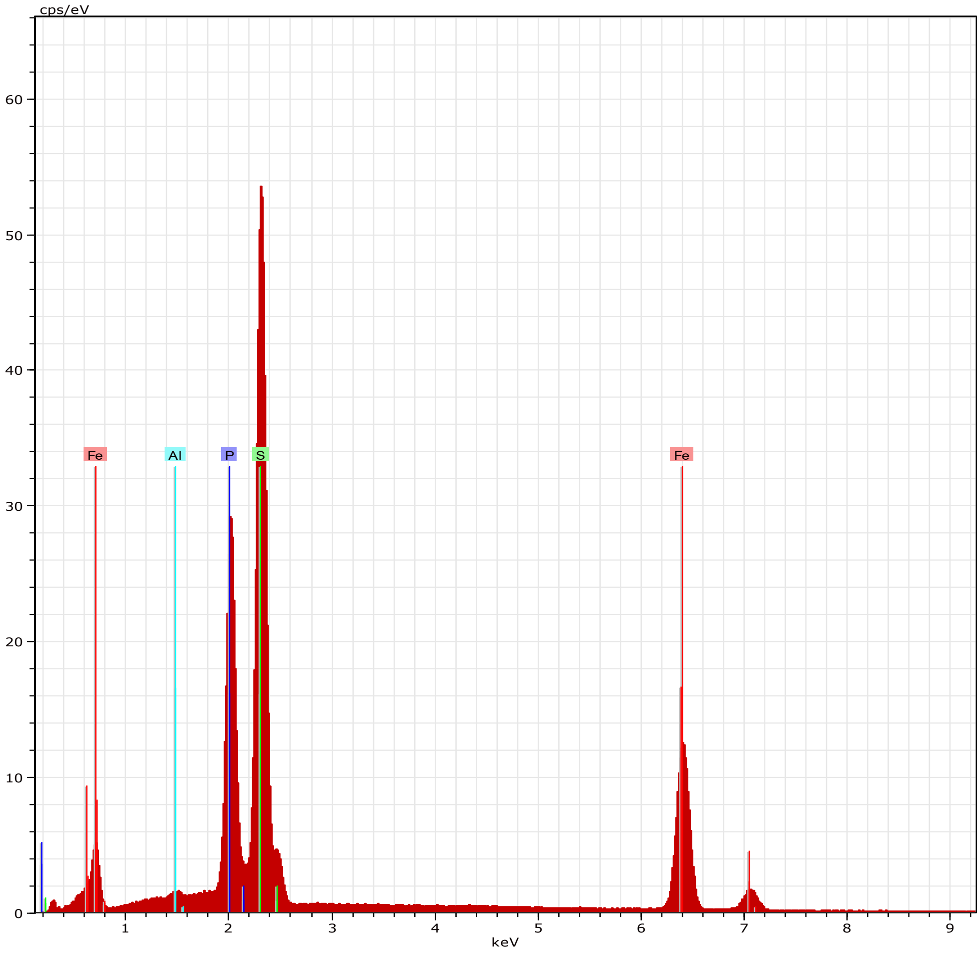

Figure S2. EDX spectrum from the ternary phase region 6.

Table S1. EDX data from the ternary region 6.

Spectrum: 6

Element	Series	unn. C [wt.%]	norm. C [wt.%]	Atom. C [at.%]	Error [%]
Iron	K-series	36,73	38,16	25,90	1,0
Sulfur	K-series	39,57	41,12	48,61	1,4
Phosphorus	K-series	19,24	20,00	24,47	0,8
	Total:	96,23	100,00	100,00	

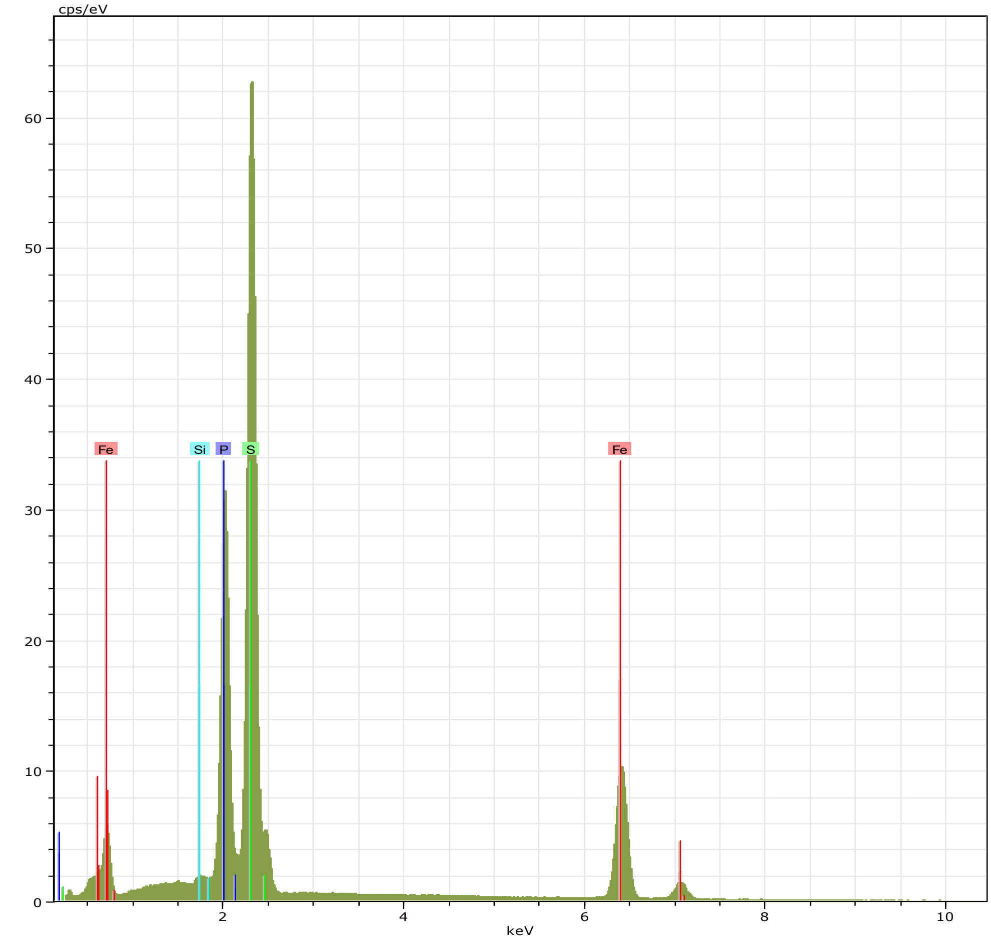

Figure S3. EDX spectrum from the ternary phase region 7.

Table S2. EDX data from the ternary region 7.

Spectrum: 7

Element	Series	unn. C [wt.%]	norm. C [wt.%]	Atom. C [at.%]	Error [%]
Iron	K-series	33,05	36,51	24,63	0,9
Sulfur	K-series	41,12	45,43	53,37	1,5
Phosphorus	K-series	16,06	17,74	21,58	0,6
Silicon	K-series	0,29	0,32	0,43	0,0
	Total:	90,51	100,00	100,00	

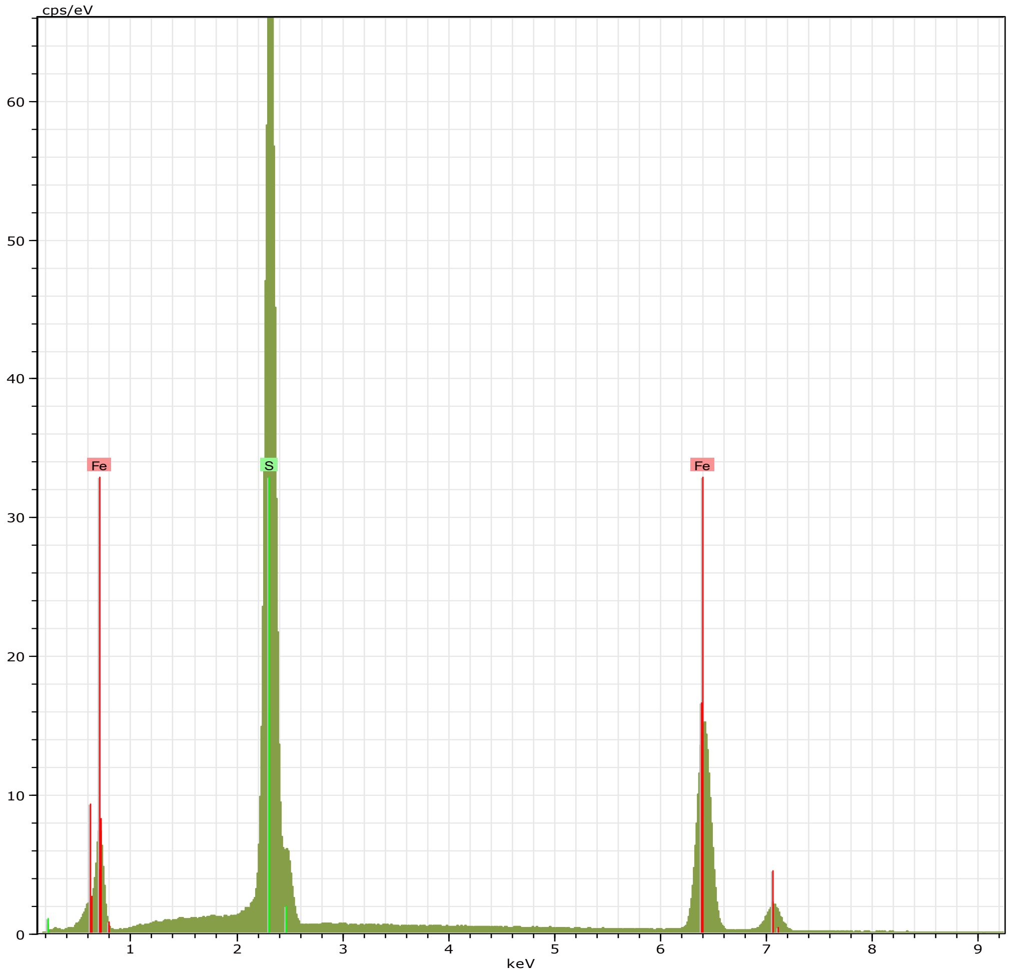

Figure S4. EDX spectrum of the pyrite FeS_2 phase. This spectrum reflects the elemental composition of the stoichiometric pyrite, crystal nr 3 in Figure S1, also discussed in multiple parts of this article.

Table S3. EDX data of the pyrite material, region 3.

Spectrum: 3

Element	Series	unn. C [wt.%]	norm. C [wt.%]	Atom. C [at.%]	Error [%]
Iron	K-series	44,72	46,76	33,52	1,2
Sulfur	K-series	50,91	53,24	66,48	1,8
	Total:	95,63	100,00	100,00	

Table S4. EDX data of the pyrite material, region 4.

Spectrum: 4

Element	Series	unn. C [wt.%]	norm. C [wt.%]	Atom. C [at.%]	Error [%]
Iron	K-series	45,77	47,37	34,07	1,2
Sulfur	K-series	50,86	52,63	65,93	1,8
	Total:	96,63	100,00	100,00	

S2 EDX mapping results of FeS_2 materials

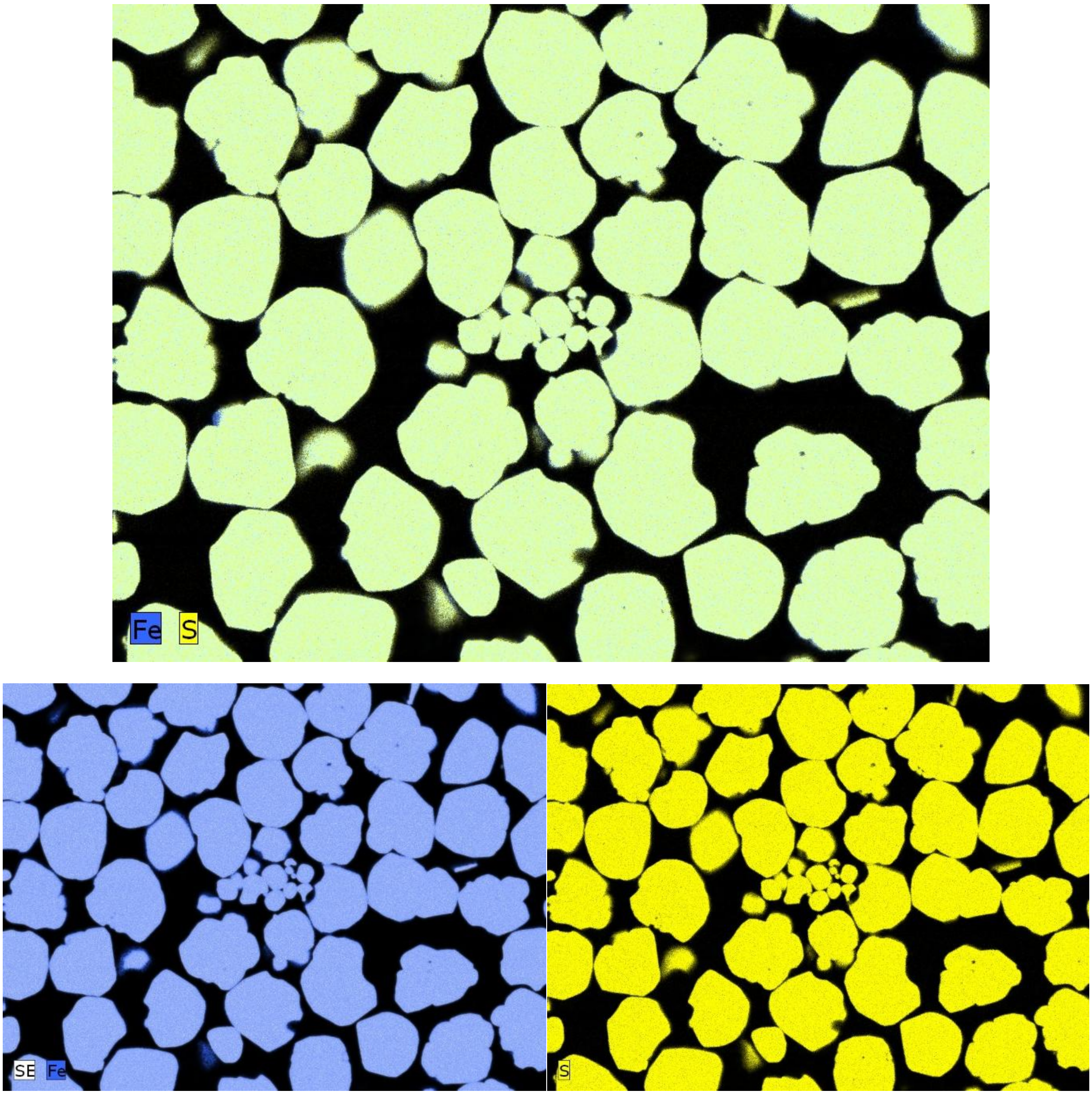

Figure S5. EDX elemental mapping of the pyrite material synthesized with 5 at% phosphorus. Similar mapping results were received in the case of materials recrystallized in 40-1000 ppm of elemental P and KI mixture.

S3 Uniformity of the material across larger batches, scalability of this method

Ensuring the uniformity of the synthesized microcrystals is critical, particularly to the reproducibility of photovoltaic device performance. The flux-growth method employed in our work has been previously utilized and well-documented by other members of our laboratory. A company that mass-produces monograin layer solar cells has also been established by our lab. This company, called Crystalsol, proved that it is possible to produce high-quality monograin powders for nearly all compound semiconductors of interest for photovoltaics [1].

Crystalsol scaled the synthesis volumes up by increasing the amounts from 30 g synthesized in sealed quartz ampoules to a few kg synthesized in graphite containers. In the flux-growth method, the composition of the microcrystals can be precisely controlled through the stoichiometry of the precursors. When the volume of liquid flux added to the ampoule is approximately equal to the volume of the solid precursors, the flux effectively separates the precursors and facilitates uniform distribution. Due to the synthesis in closed volumes, volatile vapor loss and the creation of side products can be prevented. This is otherwise a critical problem in large-area thin film module production.

Upon completion of synthesis, the microcrystals are sieved into narrow size fractions. Solar cells are fabricated from individual size fractions and show consistent performance across samples. In our current work with pyrite, we typically synthesize batches of 1–10 grams, and the resulting materials have shown a high degree of homogeneity.

[1] https://doi.org/10.1016/j.solmat.2022.112160

S4 The reaction pathway for the creation of the layered $FePS_3$ phase

The secondary $FePS_3$ phase, observed in Chapter 3.1 and in much lower amounts in Chapter 3.3, causes cracking and fragmentation of the crystals and could prevent the doping of pyrite. Based on these, we propose the following reactions between elemental phosphorus, which is found in the FeS+P material, and added sulfur, a precursor of pyrite. These reactions take place during pyrite synthesis (Chapter 3.3) and the P vapor treatment of previously prepared pyrite crystals (Chapter 3.1). During the synthesis, sulfur vapor pressure is created by the S precursor. During the P vapor treatment, sulfur vapor is created by heating FeS_2:

$$2\ FeS_2\,(s) \leftrightarrow 2FeS\,(s) + S_2\,(g) \tag{1}$$

In degassed ampoules at high temperatures, a thermodynamic equilibrium forms between solid (s) and gaseous (g) phases. As the activity of solids is equal to 1, the equilibrium for the reaction (1) can be written as

$$K_p \approx P_S \tag{2}$$

where K_p is the equilibrium constant and P_S is the pressure of sulfur. Sulfur pressure remains similar to the pressure of phosphorus until 450 °C (around 800 torr)[2]. After that, the phosphorus pressure increases much more rapidly than the sulfur pressure, reaching up to 100,000 torr at 690 °C[3]. The gaseous phosphorus reacts with sulfur to form P_2S_5 and infiltrates pyrite crystals through their dislocations:

$$P_4\,(g) + 5\ S_2\,(g) \rightarrow 2\ P_2S_5\,(l) \tag{3}$$

As sulfur is consumed via reaction (3), the process continues to generate P_2S_5 until one of the reactants is fully depleted. This intermediate, P_2S_5, then promotes the formation of the ternary $FePS_3$ phase. D.G. Chica et al.[4] reported that P_2S_5, with a melting point of 285 °C, acts as a reactive flux that readily reacts with various metals to form mono- and bimetallic layered thiophosphates of the general formula $M_{2-x}M'_xP_2S_6$. In our system, where FeS_2 is exposed to gaseous phosphorus, the reaction can proceed as follows:

$$4\ FeS_2 + 2\ P_2S_5 \rightarrow 2\ Fe_2P_2S_6 + 3\ S_2 \tag{4}$$

As a result, the crystal composition segregates into intrinsic FeS_2 and phosphorus-rich $FePS_3$ domains. However, in Chapter 3.3, the concentration of $FePS_3$ is sufficiently low that it does not dominate the structural characteristics of *p*-type pyrite, which further proves the necessity of an appropriate FeS+P source material.

[2] https://doi.org/10.1016/S0021-9614(73)80089-8.

[3] https://doi.org/10.1021/j100786a009.

[4] https://doi.org/10.1021/acs.inorgchem.0c03577.

S5 Comparison of doping techniques

Table S5. Summary of the doping strategies used and the comparison of outcomes.

	**Vapor Phase Heat-Treatment of Pre-Synthesized FeS_2 Crystals in Phosphorus**	**Recrystallization by Heat-Treatment of FeS_2 crystals in a Mixture of KI and P**	**FeS_2 Synthesis with a Phosphorus Anion-Containing Compound**
**Morphology after the treatment**	A large portion of the crystals was fractured, exhibited cracks, and had a layered structure.	No difference in the crystals' morphology before or after the treatment.	The crystals synthesized with 2, 3, 5, and 7 at% P did not have any differences in morphology compared to undoped and untreated samples. The crystals synthesized with 10 at% P showed some cracking on the surface.
**Phase composition after the treatment**	The material was split into two distinct phases: FeS_2 and $FePS_3$.	All the materials exhibited the pyrite phase.	Only the pyrite phase was detected by Raman spectroscopy; XRD detected a weak signal of $FePS_3$.
**Elemental composition after treatment**	<u>EDX results</u><br>The pyrite phase region had a Fe:S ratio of 1:2, and no P was detected.<br>The secondary phase region had a varying P-rich Fe-S-P composition.	<u>EDX results</u><br>All materials had a Fe:S ratio of 1:2. No P was detected.<br><u>ICPMS results</u><br>The undoped and untreated material had a higher P concentration (59 ppm) than after recrystallization in P.	<u>EDX results</u><br>No P was detected from the FeS_2 crystals synthesized with 2, 3, and 5 at% P. Some of the measurements of the crystals synthesized with 7 and 10 at% P contained up to 1.4 at% of P.
**Conductivity type**	*n*-type	*n*-type	*p*-type
